# Causal cardiovascular risk factors for dementia: insights from observational and genetic studies

**DOI:** 10.1093/cvr/cvae235

**Published:** 2024-11-05

**Authors:** Emilie Westerlin Kjeldsen, Ruth Frikke-Schmidt

**Affiliations:** Department of Clinical Biochemistry, Copenhagen University Hospital—Rigshospitalet, Blegdamsvej 9, 2100 Copenhagen, Denmark; Department of Clinical Biochemistry, Copenhagen University Hospital—Rigshospitalet, Blegdamsvej 9, 2100 Copenhagen, Denmark; Department of Clinical Medicine, Faculty of Health and Medical Sciences, University of Copenhagen, 2200 Copenhagen, Denmark

**Keywords:** Cardiovascular disease, Causality, Alzheimer’s disease, Mendelian randomization, Risk factors, Vascular dementia

## Abstract

The escalating prevalence of dementia worldwide necessitates preventive strategies to mitigate its extensive health, psychological, and social impacts. As the prevalence of dementia continues to rise, gaining insights into its risk factors and causes becomes paramount, given the absence of a definitive cure. Cardiovascular disease has emerged as a prominent player in the complex landscape of dementia. Preventing dyslipidaemia, unhealthy western-type diets, hypertension, diabetes, being overweight, physical inactivity, smoking, and high alcohol intake have the potential to diminish not only cardiovascular disease but also dementia. The purpose of this review is to present our current understanding of cardiovascular risk factors for Alzheimer’s disease and vascular dementia (VaD) by using clinical human data from observational, genetic studies and clinical trials, while elaborating on potential mechanisms. Hypertension and Type 2 diabetes surface as significant causal risk factors for both Alzheimer’s disease and VaD, as consistently illustrated in observational and Mendelian randomization studies. Anti-hypertensive drugs and physical activity have been shown to improve cognitive function in clinical trials. Important to note is that robust genome-wide association studies are lacking for VaD, and indeed more and prolonged clinical trials are needed to establish these findings and investigate other risk factors. Trials should strategically target individuals at the highest dementia risk, identified using risk charts incorporating genetic markers, biomarkers, and cardiovascular risk factors. Understanding causal risk factors for dementia will optimize preventive measures, and the implementation of well-known therapeutics can halt or alleviate dementia symptoms if started early. Needless to mention is that future health policies should prioritize primordial prevention from early childhood to prevent risk factors from even occurring in the first place. Together, understanding the role of cardiovascular risk factors in dementia, improving genome-wide association studies for VaD, and advancing clinical trials are crucial steps in addressing this significant public health challenge.

## Graphical Abstract

**Graphical Abstract:**
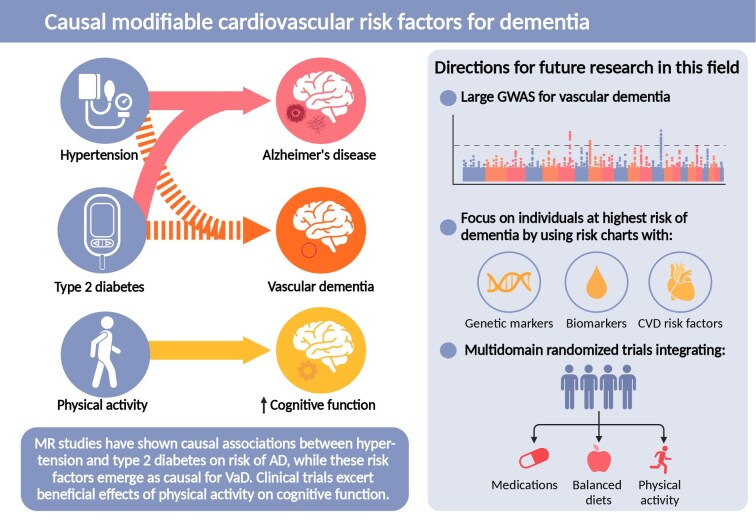
Current evidence and future research directions: causal cardiovascular disease risk factors for dementia. AD, Alzheimer’s disease; CVD, cardiovascular disease; GWAS, genome-wide association study; MR, Mendelian randomization; VaD, vascular dementia.

## 1. Introduction

The term ‘dementia epidemic’ refers to a global crisis marked by a significant increase in dementia cases.^1^ In the next 30 years, the number of people affected by dementia is expected to rise from the current 55 to 140 million worldwide, driven by expanding ageing populations.^2^ Currently, there is no effective treatment to stop dementia progression. Therefore, it is essential to identify modifiable risk factors, as nearly half of all dementia cases have the potential to be prevented, as recently estimated by the Lancet Commission 2024 update on dementia.^3^ This prevention can be accomplished by managing cardiovascular risk factors as soon as they develop. The socioeconomic impact of this crisis is substantial, underscoring the need for strategies to better prevent or slow down the disease, benefiting both human health and society.^1^

Late-onset dementia, diagnosed after 65 years of age, encompasses several subtypes. Alzheimer’s disease (AD) accounts for ∼65% of cases, vascular dementia (VaD) for about 20–30% of cases, while rarer subtypes such as Lewy body dementia and frontotemporal dementia collectively represent only a small percentage of cases.^1,2^ ‘Dementia’ is used in this review as an overall umbrella term for these subtypes. Due to frequent overlapping pathologies in dementia, vascular mechanisms often play a role in AD. It is not uncommon for individuals with AD to have co-occurring pathologies, such as cerebrovascular disease.^4^ Consequently, vascular brain health is relevant to many, including clinically diagnosed AD patients.^5^

The aim of this review is to shed light on the most common types of late-onset dementia, their pathogenic basis, and to describe important modifiable cardiovascular risk factors contributing to disease development from observational studies, Mendelian randomization (MR) studies, and clinical trials. These include HDL cholesterol, LDL cholesterol and triglycerides, unhealthy western-type diets, diabetes, hypertension, being overweight, physical inactivity, smoking, and alcohol consumption.

## 2. Dementia

Dementia is defined as a collection of symptoms, including memory loss and cognitive decline, disrupting daily life and independent function. Symptoms of dementia are divided into different stages of severity, from the early stage with forgetfulness and disorientation, to the late stage with severe memory loss, physical difficulties, and total dependence on others.^2^

### 2.1 Alzheimer’s disease

AD is a chronic neurodegenerative disease and the most prevalent dementia subtype. The diagnostic criteria include gradual onset over months to years, a clear cut history of worsening of cognition, impairment in learning and recalling recently learnt information, impairment of minimum one other cognitive domain, and exclusion of other causes.^6,7^

The heritability for AD is estimated to be as high as 60–80%^8^ and the majority of AD cases (90%) occur late in life. Uncovering genes associated with this late-onset type will facilitate the understanding of the underlying disease pathophysiology.

The most significant genetic risk factor for late-onset AD, discovered in 1993, is the ε4 allele of the apolipoprotein E (*APOE*) gene.^9^ ApoE is the major cholesterol transporter in the central nervous system (CNS),^10^ and studies suggest that apoE is involved in amyloid-β processing, tau phosphorylation, and blood–brain barrier permeability.^11^ Since 1993, over 70 genetic loci linked to late-onset AD have been identified primarily through genome-wide association studies (GWASs) that include SNPs.^12–14^ Over two dozen genetic studies on AD can be found in the GWAS catalogue.^15^ The latest, most powerful AD GWAS from the European Alzheimer’s & Dementia Biobank revealed via pathway analysis that the most significant gene sets are linked to amyloid and tau, cholesterol metabolism, endocytosis/phagocytosis, and immunity.^14^ In-depth reviews on the genetics of AD are readily available in other publications.^16,17^ Polygenic risk scores, which are developed using multiple genetic variants, hold promise in identifying individuals who are at risk of developing dementia. One such score, a weighted polygenic risk score based on 39 variants at GWAS significance and stratified by *APOE* genotype, revealed a 4–5.5 years difference in median onset of AD in *APOE*ε4 carriers.^18^ This finding highlights the potential of polygenic risk scores in identifying individuals at highest risk who will benefit the most from early prevention.^19^

**Figure 1:**
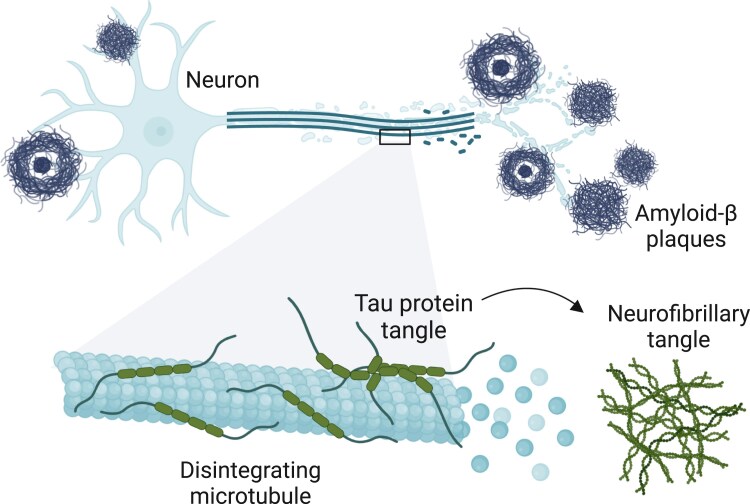
Hallmarks of AD pathology. Amyloid-β plaques are formed from Aβ-oligomers aggregation. Tau protein tangles and form neurofibrillary tangles.

The neuropathological changes that characterize AD include deposition of amyloid-β containing plaques (from Aβ40 and Aβ42 monomers) and neurofibrillary tangles of tau (*Figure 1*).^20^ Additionally, disruption in the synaptic balance is observed along with loss of neuronal density and degradation of the neuronal network integrity.^20^ AD is characterized by a prodromal or a pre-clinical phase where obvious neuropathological changes have already set in, but the patient has only slight or no declines in cognitive performances.^21^ The prodromal phase can last 15–20 years until symptoms of dementia manifest.^21,22^ Besides clinical assessment and cognitive testing, the diagnosis of AD can be supported by cerebrospinal fluid markers such as low amyloid-β_42_ levels or elevated tau levels; by a positive positron emission tomography (PET) amyloid imaging; or moreover, by decreased uptake of ^18^fluordeoxyglucose in the temporoparietal cortex detected by a PET scan.^7^ Importantly, an update to the 2018 National Institute on Aging-Alzheimer’s Association Alzheimer’s research framework suggests new diagnostic criteria for AD.^23^ These criteria involve systematically integrating blood-based biomarkers, which have displayed promising abilities and effectiveness in detecting AD.^23^ Diagnosing AD should thus be biologically defined rather than solely based on a clinical syndrome.

There are currently three cholinesterase inhibitors available for treatment of AD, and the effect on symptom progression is limited.^20^ They work by decreasing the breakdown of acetylcholine in the synaptic cleft thereby leaving the active neurotransmitter to function for a longer time.^20^ Several Phase 3 trials for other drugs have failed, which could be due to the challenges of treating sufficiently early because of the prodromal phase of AD.^20^ In 2021, the American Food and Drug Administration (FDA) granted approval for Aducanumab, a monoclonal antibody targeting amyloid-β plaques.^24^ However, the two Phase 3 trials that led to this FDA approval did not conclusively demonstrate a significant positive impact on cognitive function. In 2023, the FDA also approved Lecanemab, another monoclonal antibody that binds to soluble amyloid-β protofibrils.^25^ During a Phase 3 trial, Lecanemab exhibited a modest reduction in the decline of clinical measures related to cognition and function over 18 months, although it was associated with adverse events. Most recently, another monoclonal antibody, Solanezumab, targeting monomeric amyloid, did not slow down cognitive decline in pre-clinical AD patients in a Phase 3 trial of 1169 individuals.^26^

Many drug trials aimed at reducing or minimizing amyloid burden have not yielded the desired results, and it remains unclear whether this is due to the timing of treatment or whether amyloid plaques themselves are not the sole cause of AD.

### 2.2 Vascular dementia

VaD or major vascular cognitive impairment is defined by significant deficits in at least one cognitive domain with severe disruption of activities of daily living and evident cerebrovascular aetiology.^1,27^

A recent GWAS including 800 000 individuals and 9000 VaD cases identified known AD loci for VaD as well as novel loci associated with hypertension, diabetes, and neuron maintenance, providing support of vascular mechanisms in dementia development.^28^

**Figure 2:**
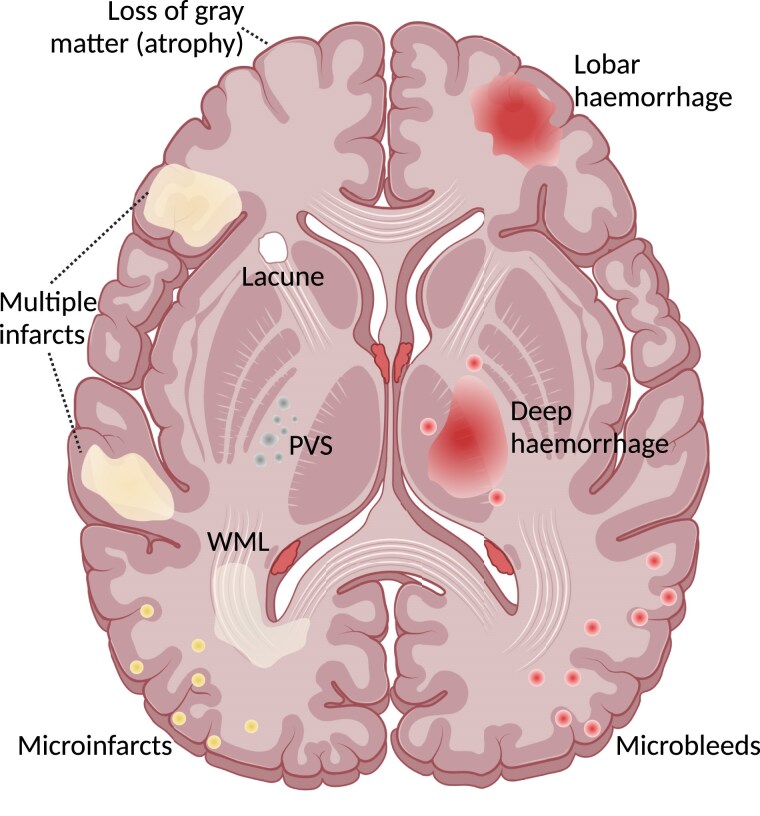
Manifestations of VaD. Horizontal section of the brain. WMLs show up as white matter hyperintensities on MRI. PVS, perivascular spaces; WML, white matter lesion.

Both small and large vessel disease are important for VaD development. Small vessel disease is a term that includes pathological findings such as micro and small infarcts, microbleeds, and atherosclerosis.^1^ Large vessel disease includes large infarcts from for example atherosclerosis in intra- and extracranial large vessels causing, i.e. stroke (*Figure 2*).^1^ Pathological changes leading to vascular cognitive impairment may be caused by a chronic hypoperfusion of the brain tissue.^1^ This continued reduction in cerebral blood flow produces white matter injury, brain atrophy, infarcts, and haemorrhages.^29^ Some of these pathological changes are also observed in AD.^1^ It remains unclear whether the reduced cerebral blood flow is a consequence or a causative factor of decreased metabolic demands.^1^

In contrast to the more slowly progressing AD, the onset of vascular cognitive impairment is often more acute with an unpredictable course and can progress in a stepwise manner. Patients with vascular cognitive impairment often present with deteriorations in processing speed and executive functions, while the appearance of memory loss is not a requirement.^1^ However, symptoms of vascular cognitive impairment are in general much more heterogeneous than for AD. Diagnosing VaD includes a clinical assessment, cognitive testing, neuroimaging evidence of cerebrovascular disease, and testing for cardiovascular risk factors, including a blood lipid profile and blood glucose. Imaging manifestations of VaD comprise of white matter hyperintensities, lacunes, cerebral microbleeds, atrophy, perivascular spaces, large infarcts, and large haemorrhages (*Figure 2*).^1^ Treatment with cholinesterase inhibitors can be used; however, only small benefits on cognition have been demonstrated in randomized controlled trials.^1,29^ Addressing comorbidities and the underlying vascular aetiology are key treatment components for VaD as of today.^30^

While research on VaD is less extensive compared with AD, key risk factors have been identified to associate with VaD. However, the mechanisms underlying VaD are still unclear, and the development of effective treatments is limited.^1^ VaD deserves intensified focus as this is the subtype of dementia that most likely will be preventable by addressing cardiovascular risk factors.

## 3. Modifiable cardiovascular risk factors for dementia

Dementia is a complex disease caused by a range of interacting risk factors. The investigation of modifiable risk factors is challenging because individuals in observational studies and clinical trials may already have advanced underlying disease without the appearance of symptoms at inclusion. Furthermore, the identification of critical risk factors in midlife poses a challenge for both observational studies and randomized controlled trials, given the necessity for long follow-up periods, which are necessary to determine whether an intervention or risk factor is associated with dementia, debuting decades later. In clinical trials, it is challenging to identify individuals who are at risk of developing dementia but are still in the pre-prodromal and pre-symptomatic stage. However, this phase represents an optimal time to recruit individuals for inclusion, since neurodegenerative processes are likely still in reversible stages. Observational studies, while useful, hold the risk of reverse causality and confounding, and can therefore not in themselves confer causality. To overcome these challenges from clinical trials and observational studies, MR utilizes randomly assorted genetic variants, as unconfounded proxies for modifiable exposures of interest, to suggest causal contributions of a risk factor to disease, reflecting lifetime impact.^31–33^

### 3.1 Lipids and lipoproteins

There are four major lipoprotein classes in plasma constituting of HDL, LDL, very LDL (VLDL), and chylomicrons. The role of LDL, VLDL, and chylomicrons, which all contain apolipoprotein B (apoB), is to distribute triglycerides and cholesterol to peripheral tissues.^34,35^ HDL is produced in the liver by initial lipidation of apolipoprotein A1 (apoA1) from phospholipids to form a pre-β HDL particle.^36^ This HDL particle further matures by taking up cholesterol and phospholipids from peripheral tissues, such as adipose tissue, to form a small HDL particle. Different lipases and transferases further mature and remodel the HDL particle. The HDL-derived cholesterol esters are taken up by the scavenger-receptor B1 (SR-B1) in the hepatocyte, whereas cholesterol and triglycerides from apoB-containing lipoproteins are taken up by the LDL receptor.^36^ In the brain, cholesterol metabolism is isolated from the peripheral circulation due to the blood–brain barrier (BBB). ApoB-containing lipoproteins are absent, as the CNS does not use triglycerides as a source of energy. Consequently, the transport of lipids in the brain is dependent on HDL-like particles.^37^ ApoE is the most abundant HDL protein in the brain, whereas apoA1 is the most abundant in the periphery.^37,38^ ApoA1 is transferred across the BBB, and discoidal apoA1-containing particle uptake has been shown to be mediated by SR-B1 transcytosis or cholesterol-mediated endocytosis.^39,40^

#### 3.1.1 Plasma HDL cholesterol concentrations

Observational findings regarding circulating HDL cholesterol concentrations and cognitive impairment or dementia are inconsistent.^41–51^ In prospective studies, both low and high levels of HDL cholesterol have been reported to be associated with the risk of dementia and AD,^41,44–49^ and most recently, a cohort study including 184 367 individuals found a U-shaped association between plasma HDL cholesterol concentrations and the risk of AD-related dementia.^52^ Interestingly, one study published that a specific HDL subclass (the ratio between cholesterol ester concentration to total lipids in large HDL particles) was associated with higher dementia risk in eight prospective cohorts totalling 22 623 participants.^46^ In a study, including two prospective cohorts of 111 984 individuals combined, very high concentrations of plasma HDL cholesterol were associated with a high risk of both AD and dementia, but not with vascular-related dementia.^53^

AD has been the main outcome of investigation in MR studies and most of them have not found any association between genetically proxied elevated HDL cholesterol concentrations and AD risk when applying sensitivity analyses or polygenic risk scores.^53–56^ In contrast, Luo *et al.*^57^ recently published a two-sample MR study using the largest consortia to date including 400 000 controls and 39 000 cases of AD. They found novel genetic associations between high HDL cholesterol concentrations and risk of AD using multivariable analyses. The genetic instruments used for HDL cholesterol concentrations marked well-known genes for HDL cholesterol biology. However, studies using individual level data are needed to confirm this association. So far, there are no MR studies investigating HDL cholesterol concentrations and their genetic association with vascular-related dementia.

The mechanisms behind potential influences from circulating HDL cholesterol on brain HDL-like metabolism and AD pathology are unknown. However, since peripheral apoA1 and small discoidal HDL cholesterol can enter the brain tissue via the BBB or the blood cerebrospinal fluid barrier,^58,59^ this could impact the metabolism of the HDL-like particle in the brain. It has been shown that a mutation in the gene encoding SR-B1 results in high HDL cholesterol concentrations with more buoyant and larger HDL particles.^60,61^ Thus, when peripheral HDL cholesterol concentrations are high, HDL particles may become defective in supplying the brain with apoA1 and other beneficial molecules because they cannot enter the BBB. This could lead to less maturation of the HDL-like particle in the brain, thereby compromising normal cerebral lipid transport and amyloid-β clearance.

Given recent studies linking high HDL cholesterol concentrations with dementia and AD, it will be interesting to explore these findings further by investigating HDL particle subtypes, including their size and function, for dementia risk and pathology.

#### 3.1.2 Plasma triglyceride concentrations

Plasma triglycerides are markers of triglyceride-rich lipoproteins, and causally linked to atherosclerotic cardiovascular disease due to their cholesterol content.^62^ In prospective studies in recent years, findings have shown that high concentrations of circulating triglycerides are associated with an increased risk of cognitive decline and VaD or vascular-related dementia.^63–67^ In general, observational studies have not found a link between circulating triglycerides and AD risk.^63,65,66^ MR studies investigating genetically proxied triglyceride levels and risk of dementia are sparse, and so far, no association between genetically proxied elevated triglyceride concentrations and risk of AD has been found,^54,56,57^ and the need for larger studies covering VaD, is apparent.

Mechanistically, it is plausible that high plasma triglyceride concentrations (marking triglyceride-rich lipoproteins that, due to their cholesterol content, are atherogenic) could be a risk factor for VaD.^62^ When concentrations of elevated triglycerides are mild to moderate, triglyceride-rich lipoproteins can in fact enter the arterial wall as they are sufficiently small.^68^ This can lead to entrapment and accumulation of these lipoprotein types consequently causing atherosclerosis.^62,68^ Sequentially, this may initiate brain hypoxia due to hypoperfusion from narrowed arteries, ultimately causing VaD.

Taken together, there is likely no causal association between high triglyceride concentrations and AD. MR studies are warranted regarding VaD given the observational findings. For now, triglyceride concentrations can potentially be used as a biomarker for VaD risk.

#### 3.1.3 Plasma LDL cholesterol concentrations

The most cholesterol-rich lipoprotein in plasma is LDL cholesterol, and the majority of the total cholesterol content in plasma comes from LDL cholesterol. A meta-analysis of 1.2 million individuals identified high concentrations of LDL cholesterol in midlife to be associated with increased risk of all-cause dementia.^69^ Moreover, a meta-analysis of observational studies suggested statins may protect against dementia and AD; however, randomized trials have not shown efficacy on cognitive decline.^70–72^

Most MR studies have not observed associations between genetic proxied elevated LDL cholesterol concentrations and AD risk.^54–57^ Interestingly, one study found that variants associated with low LDL cholesterol levels, which mimic the effects of lipid-lowering therapy, were linked to a reduced AD risk.^73^ These findings suggest that lipid-lowering therapy could potentially help prevent AD. Nevertheless, it is worth noting that these studies did not perform sensitivity analyses excluding Chromosome 19, addressing the independence of the strong *APOE* locus. Observational and genetic studies regarding LDL cholesterol and VaD are sparse. Yet, one small MR study discovered a link between genetically proxied high LDL cholesterol concentrations and significant attributes of cerebral small vessel disease, a significant precursor to VaD.^74^

LDL cholesterol could be a causal risk factor for dementia explained by its well-known atherogenic effects.^35,75^ The key biological mechanisms of this primary atherogenic driver include influx and retention of the LDL particle into the arterial intima wall, which leads to the formation of foam cells from macrophages taking up LDL particles, release of proinflammatory lipids, activation of the innate and adaptive immune response, all of which contribute to plaque formation.^75^

LDL cholesterol has been added as one of the 14 modifiable risk factors for dementia in the newest Lancet Commission report.^3^ However, the causal association between LDL cholesterol concentrations and AD remains unclear while studies on VaD are lacking. Comprehensive individual level data MR studies are warranted for both AD and VaD, addressing the strong pleiotropic contribution from *APOE*.

### 3.2 Diet

Our understanding of how diet affects dementia and cognition is still developing. Furthermore, diet is not a single factor but rather a complex, multi-dimensional exposure involving various healthy and unhealthy elements present in our food and drink choices, influenced by specific habits, and often intertwined with other aspects of our lifestyle.^76^ In recent years, observational studies obtaining information on nutrition and dementia have shifted from investigating specific macronutrients or food components to cover overall dietary habits.^77^ This change is partly due to the difficulty in isolating specific macronutrients or ingredients because of confounding factors. For example, individuals with a low intake of vegetables often have higher intakes of ultra-processed foods or red meats, making it difficult to distinguish the effects of individual dietary components.

Prospective observational studies have extensively investigated the connection between diet and dementia, excluding VaD, the latter remaining more understudied.^78^ The majority of observational studies, despite their heterogeneity, find overall healthy diets to be associated with reduced risk of cognitive decline, all-cause dementia, and AD as also reported in systematic reviews and meta-analysis.^78–80^ There is limited data from randomized clinical trials regarding the influence of healthy diets on cognitive decline, making it challenging to draw definitive conclusions about their efficacy.^81^ In a recent randomized controlled trial led by Barnes *et al.*,^82^ they discovered that among older individuals without cognitive impairment but with a family history of dementia, there were no significant disparities in cognitive changes over a 3-year span between those who adopted the MIND diet (The Mediterranean–DASH Intervention for Neurodegenerative Delay) and those who adhered to a control diet with mild caloric restriction. The lack of significant findings could be partly due to the relatively short trial period of 3 years, or due to the control-diet group experiencing similar weight loss as the intervention group. Sparce evidence exists from studies examining overall dietary habits and VaD or vascular-related dementia.^78,83–85^ One study of 94 184 individuals found increased risk of vascular-related non-Alzheimer’s dementia for those with low adherence to dietary guidelines, independent of cardiovascular risk factors and *APOE* genotype.^83^ However, more observational studies are needed to conclude whether diet is associated with VaD, and randomized controlled trials are vital to estimate causality. To date, MR studies exploring the causal links between genetically proxied dietary factors or nutrients and dementia have not yielded convincing findings.^86,87^ Moreover, using diet as a genetic instrument in MR studies poses a particular challenge as the genetic variant associated with diet should not share any common causes with the dementia outcome or associate with confounders, as this would violate the MR assumptions.

Low adherence to dietary guidelines could impact the risk of dementia via several pathways. It is well known that poor western-type diets (high in saturated fat and meat, and low in vegetables, fruits, grains, and unsaturated fats) cause hyperlipidaemia contributing to atherosclerosis which could affect risk of dementia.^88^ Moreover, these types of diets may lead to obesity, hypertension, and Type 2 diabetes, which could then be potential mediators for increased dementia risk.^89,90^ Of all risk factors, diet has the greatest potential to prevent obesity, hypertension, diabetes, and dyslipidaemia.^91^ It is therefore essential to place greater emphasis on diets that promote cardiovascular health, highlighting implementation strategies and achievable dietary changes.^76,92–94^

Most observational studies point towards associations between western-type diets and risk of both AD and VaD; however, further confirmation for VaD is warranted. To test causality, randomized controlled trials should be carried out for longer periods and include individuals in pre-symptomatic phases with high risk of developing dementia.

### 3.3 Hypertension

Multiple meta-analyses of observational studies have found that midlife hypertension, specifically systolic blood pressure over 140 mmHg, is associated with an increased risk of AD and cognitive impairment, with stronger associations in midlife than late life.^90,95^ Furthermore, a large meta-analysis including both prospective observational studies and randomized controlled trials, assessed several risk factors for AD, and found that midlife hypertension was associated with a relative risk of 1.38 (1.29–1.47) for AD.^96^ As for VaD, observational findings from a meta-analysis point towards an increased risk from hypertension, yet, updated studies including more cases are warranted.^97^ Meta-analyses including observational and/or randomized clinical trials have shown that individuals treated with anti-hypertensive medication, have a reduced risk of developing all-cause dementia and AD compared with untreated individuals, regardless of the type of medication used.^98–101^ Low blood pressure has also been associated with increased dementia risk, especially in older ages, yet, this is likely due to reverse causation.^102^

MR studies have shown mixed results on the relationship between genetically proxied hypertension and AD. Earlier studies found either no association or a lower risk between genetically proxied raised systolic blood pressure and AD.^54,103,104^ However, genetically proxied raised systolic blood pressure was also associated with higher likelihood of using anti-hypertensive treatment.^54^ A recent multivariable MR study by Luo *et al.*^57^ robustly demonstrated that proxied high systolic blood pressure was associated with higher AD risk, using data from the largest AD consortia to date. An MR study by Taylor-Bateman *et al.*^74^ investigating features of cerebral small vessel disease found that both genetically proxied elevated systolic and diastolic blood pressure were associated with increased white matter hyperintensities, decreased fractional anisotropy and increased mean diffusivity, the latter two characterizing magnetic resonance imaging (MRI) microstructural changes in cerebral small vessel disease. This supports that hypertension may be an important causal risk factor for VaD.

**Figure 3:**
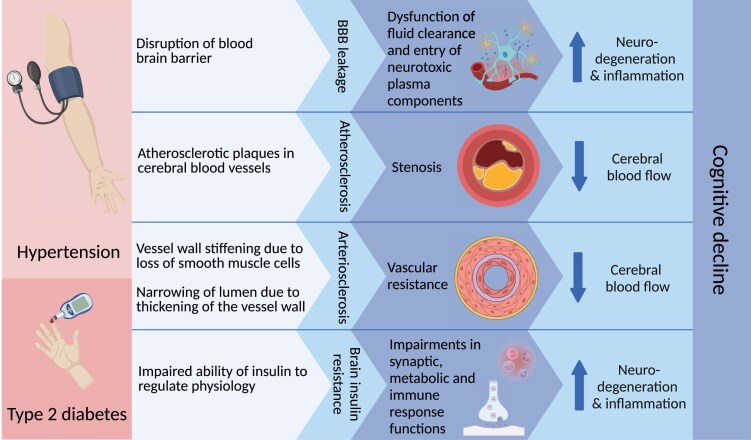
Potential mechanisms linking hypertension and Type 2 diabetes to dementia pathology. This figure illustrates proposed mechanisms by which hypertension and Type 2 diabetes may contribute to dementia pathology. Although the exact pathways are not fully established, potential mechanisms are depicted. For instance, in Type 2 diabetes, it is unknown which factors such as hyperglycaemia, hyperinsulinaemia, and hypertension contribute to brain insulin resistance and cognitive impairment.^1,105–109^.

Currently, the totality of evidence points towards an association between midlife hypertension and future risk of several dementia subtypes. Mechanistically, elevated blood pressure can lead to vessel wall stiffening as well as the promotion of atherosclerosis in the arteries of the brain which together may result in cerebral hypoperfusion (*Figure 3*).^1,105–107^ Hypertension can also lead to disruption of the BBB causing leakage and thus dysfunction in the clearance and entry of neurotoxic components.^107^ Moreover, hypertension is important in the development of white matter lesions and lacunar infarcts in cerebral small vessel disease, as well as in the formation of large infarcts and thus stroke in large vessel disease.^105^

### 3.4 Diabetes

Meta-analyses of observational studies have consistently found that individuals with Type 2 diabetes have increased risk of dementia, including AD and VaD.^110,111^ Moreover, a more recent prospective registry-based study including 784 434 individuals found Type 2 diabetes to be associated with AD, VaD, unspecified dementia, as well as all-cause dementia.^112^ A meta-analysis of observational studies of anti-diabetic drugs on dementia risk showed that dipeptidyl peptidase-4 inhibitors were the most effective anti-diabetic medication in mitigating dementia risk among diabetic patients.^113^ This was followed by metformin and thiazolidinedione. Studies of glucagon-like peptide (GLP)-1 receptor agonists and sodium glucose co-transporter-2 inhibitors were not included as studies were absent.^113^ In a pooled analysis of randomized controlled trials, patients with diabetes randomized to GLP-1 receptor agonists had lower rates of dementia compared with those randomized to placebo.^114^ In the same study, this reduced dementia risk was also observed when analyses were conducted in a nationwide prospective cohort.^114^

Several MR studies have failed to identify significant associations between genetically proxied elevated glucose levels or diabetes and the risk of AD.^54,57,103,112,115,116^ However, a few MR studies investigating the impact of genetically proxied effects of different anti-diabetic drug classes and risk of AD were shown to lower odds of AD.^117,118^ Moreover, Type 2 diabetic parameters such as genetically proxied higher fasting glucose concentrations and lower HOMA-β-cell function were associated with higher risk of AD in a two-sample MR.^119^ The most recent one-sample MR, using the Million Veterans Program, demonstrated that genetically proxied diabetes was associated with all-cause dementia, AD, and VaD.^120^ While the earlier studies did not reach statistical significance, the effect strength and direction for AD are similar between these and the most recent study.^112,116,120^ The one-sample MR had access to individual level data and multiple diagnoses of dementia, which could likely explain their ability to identify evidence of causality, while two-sample MR rely on GWAS summary data, thus currently limited to AD alone. Notably, the Million Veterans Program consists mainly of men and to generalize these findings, large individual level studies need to be performed in women and in different ethnicities.

Vascular risk factors associated with Type 2 diabetes dispose to micro- and macrovascular complications in the CNS and especially to cerebral small vessel disease, which significantly increases the risk of VaD (*Figure 3*).^121^ Thus, damage to the cerebrovascular system, like in cerebral small vessel disease including arteriosclerosis, may be the link between Type 2 diabetes and in particular VaD. The biological mechanism between Type 2 diabetes and AD remains widely unknown; however, one hypothesis suggests that a disrupted insulin signalling is involved (*Figure 3*).^108,122^

Taken together, the current evidence suggests a causal association between Type 2 diabetes and both AD and VaD. However, the inconsistency in results underscores the complexity of the relationship. Controlled trials testing anti-diabetic drugs could reveal effects on dementia symptoms. Two current undergoing clinical trials, evoke and evoke+, are testing the GLP-1 receptor agonists semaglutide’s effect in patients with early AD with one of the trials further including participants with small vessel co-pathology.^109^ This type of medication has substantial impact not only on diabetic parameters but also on weight loss and downstream factors like improved lipid profiles and blood pressure.^123,124^ Improvement of these parameters could directly affect dementia risk.

### 3.5 Overweight and obesity

The relationship between being overweight and obese and dementia risk is complex. A large meta-analysis of cohort studies found lower BMI associated with higher all-cause dementia risk but did not differentiate between midlife and late-life BMI.^125^ Similar findings have been shown for non-VaD (mainly AD) in a separate meta-analysis.^126^ However, a different meta-analysis revealed that midlife overweight/obesity (age <65 years) was associated with increased dementia risk, while late-life overweight/obesity (age >65 years) with decreased risk.^127^ Inverse associations may indicate reverse causation when weight was assessed in late life. Being underweight late in life is often linked to underlying health issues and decreased appetite leading to weight loss, which could be early indicators of dementia prior to an official diagnosis. High waist circumference, compared with low waist circumference has been associated with a 10% increased risk of cognitive impairment and dementia.^128^ For VaD, one meta-analysis of 2.8 million individuals and 57 294 cases showed a u-shaped risk pattern for VaD with increased risk for individuals who were underweight or obese.^126^

Recently, several MR studies have been conducted to evaluate a potential causal association between BMI and AD risk, and most studies found no association.^54,103,129–131^ One MR study observed that high BMI was associated with lower risk of AD.^57^ However, individual level data suggest that these findings are limited to older age groups,^132^ illustrating that survivor bias is a major issue in summary level two-sample MR studies of diseases of old age, AD being the classical example. These MR studies are genetic case–control studies, where the cases who survive to be included tend to have fewer cardiovascular risk factors. As a result, these studies often produce counterintuitive MR results because this ‘survivor group’ has a lower prevalence of cardiovascular risk factors. For VaD, two small MR studies evaluating genetically proxied higher waist-to-hip ratio and BMI, respectively, found associations for increased risk of white matter hyperintensities, an important feature of cerebral small vessel disease.^74,133^ One of these studies showed that the effect on white matter hyperintensities was partially mediated via systolic blood pressure, suggesting that hypertension could explain the mechanism for these findings.^133^

The relationship between being overweight/obese and dementia remains unclear. Future research should prioritize distinguishing between midlife and late-life stages.

### 3.6 Physical inactivity

A recent meta-analysis of 58 prospective cohort studies with at least 1 year of follow-up and including individuals without known cognitive impairment at baseline found pooled effects between high physical activity and decreased risk of both all-cause dementia, AD and VaD.^134^ Similar results were found when only assessing studies with >20 years of follow-up, which is important to consider given the plausibility of reverse causation.^134^ Another meta-analysis of cohort studies observed a linear dose–response relationship between physical activity levels and lower risk of all-cause dementia and AD.^135^ Moreover, low physical activity levels appeared to be associated with 60% increased risk of vascular-related dementia compared with individuals with high physical activity levels in 117 616 individuals.^136^ A meta-analysis from 2016 of 18 physical activity intervention trials found a positive effect of physical activity on cognitive function.^137^ It is crucial to conduct longer intervention trials to evaluate the potential impact of physical activity on cognitive function and dementia outcomes. Additionally, these trials should explore the effects of various types and intensities of physical activities.

MR studies have investigated the link between genetically proxied physical activity and AD risk, however, with only few findings. One study observed that genetic proxies for walking were associated with lower risk of AD in a two-sample MR using the UK Biobank for the instrumental variables.^138^ No associations were observed for overall physical activity, moderate-intensity activity or sedentary behaviour.^138^ Another MR study, also using the UK Biobank for genetic instruments of physical activity but from accelerometers, found no association with AD.^139^ These findings and studies highlight the need for better physical activity GWASs, as instrumental variables for this exposure currently suffer from pleiotropy and weak instrument bias.

Physical activity may directly contribute to an anti-inflammatory response, inhibit neurodegeneration, improve endothelial function and DNA repair in prevention of cognitive impairment.^140^ Additionally, physical activity could potentially prevent dementia indirectly via beneficial effects on blood pressure, plasma lipids and lipoproteins, and perhaps via diabetes and obesity.^136^

### 3.7 Smoking

Smoking is associated with the risk of dementia as shown in observational studies, however, with inconsistent results from MR studies. Several meta-analyses of prospective studies find smoking to be a risk factor for dementia, AD and VaD with increased risks ranging from 13 to 40%.^141–143^

For two-sample MR studies, inverse associations have been found suggesting genetically proxied quantities of smoking lowers AD risk.^54,57,103^ These counterintuitive associations are likely due to survival bias inherent in two-sample MR studies of diseases of very old age. In contrast, a one-sample MR study found a trend towards genetically proxied smoking and higher risk of all-cause dementia and AD in a more genetically homogenous population.^144^ A meta-analysis with pooled results from two summary-statistics-based MR studies did not estimate any association between smoking and AD.^145^ For VaD, one MR study found that genetically proxied smoking initiation was associated with features of cerebral small vessel disease.^74^

Mechanistically, smoking is suggested to impact dementia via vascular disease, as smoking is known to accelerate atherosclerosis.^146^ Moreover, smoking induces oxidative stress which may lead to inflammatory responses which can affect dementia pathology.^146^

### 3.8 Alcohol consumption

Excessive alcohol consumption is a well-known risk factor for, in particular, alcohol-related dementia. However, observational findings for late-onset dementia are inconsistent.^147^ Several observational studies have reported an inverse association between midlife moderate alcohol consumption and dementia risk compared with abstinent drinkers. These findings could be reasoned by survivor bias or due to former risk drinkers being included in the abstinent group.^147–149^ Nevertheless, findings also show that excessive alcohol consumption above the recommended weekly intake is associated with increased risk of dementia.^149,150^ So far, no MR studies have found any causal relations between genetically proxied alcohol consumption including moderate alcohol intake and late-onset dementia risk.^57,103,151^

## 4. Multidomain clinical trials

Multidomain interventions targeting diet, physical activity, and cognitive training have shown conflicting results.^76,152–155^ The Prevention of Dementia by Intensive Vascular Care trial aimed to reduce cardiovascular risk factors over 6 years but did not demonstrate an overall beneficial effect on dementia.^152^ Similarly, the Multidomain Alzheimer Preventive Trial showed no cognitive benefits over a 3-year period.^155^ Potential reasons for these outcomes include inadequate trial duration, suboptimal participant age, or insufficient targeting of interventions. In contrast, the Finnish Geriatric Intervention Study to Prevent Cognitive Impairment and Disability trial reported improved cognition after 2 years through dietary changes, cognitive training, and exercise.^153,154^ These results may be attributed to the specific outcomes measured, high participant compliance, or a broader age range in the study. Future trials should focus on high-risk populations for dementia, potentially identified through risk charts incorporating genetic markers in polygenic risk scores, biomarkers, and modifiable risk factors.^19,156^ Emerging pharmaceuticals, such as GLP-1-receptor agonists, have shown substantial influence on multiple domains, such as reduced dietary intake, weight loss, lower blood pressure, lower cholesterol levels, and diabetes indicators, including lower haemoglobin A_1c_ and fasting plasma glucose concentrations.^123,124^ Considering the impact of these drugs on cardiovascular risk factors, it is plausible that they could also affect the risk of dementia. Results from the evoke trials, which are evaluating oral semaglutide in early AD patients, are anticipated to be available in 2025.^109^ It is crucial to note that while these medications reduce food intake, they may not necessarily alter dietary habits herein quality. Consequently, the consumption of unhealthy western diets may persist. Therefore, comprehensive trials encompassing, e.g. GLP-1-receptor agonists, balanced diets, and physical activity interventions are essential to deduce causality and effects on dementia outcomes.

## 5. Conclusions and perspectives

Dementia, particularly AD and VaD, is a significant global concern. VaD, comprising 20–30% of all dementia cases, warrants more attention and robust GWASs to explore genetic pathways and modifiable risk factors. Hypertension emerges as a significant risk factor for both AD and VaD, with MR studies illustrating its causal association with AD and suggesting an equivalent contribution to VaD. Type 2 diabetes, another key risk factor, is associated with AD through robust evidence from observational and MR studies and is emerging as a potential risk factor for VaD. Medications targeting diabetes and obesity have demonstrated beneficial effects on cardiovascular risk factors that may influence dementia risk. Physical inactivity is an important risk factor for both AD and VaD, with clinical trials indicating beneficial effects of physical activity on cognitive function. Comprehensive trials integrating medication, balanced diets, and physical activity are necessary to establish causality and determine their impact on dementia outcomes. Future research should focus on individuals at the highest risk, using risk charts that include genetic markers, biomarkers, and cardiovascular risk factors. This approach ensures that preventive measures and treatments are directed where they are needed and can benefit the most. The growing concern of dementia on a global scale urgently calls for a multifaceted approach integrating robust scientific research, targeted interventions, and comprehensive health policies.

## Authors’ contributions

E.W.K. and R.F.-S. conceived and defined the scope of the review article, and reviewed and edited the manuscript for clarity and accuracy. E.W.K. conducted the literature review, identified relevant studies, extracted data; drafted the initial version of the manuscript; and designed the figures and tables presented in the manuscript. R.F.-S. provided supervision and guidance during the entire review project. All authors critically reviewed the manuscript and approved it for submission.

## Data Availability

No new data were generated or analysed in support of this review.
